# Robot Object Detection and Tracking Based on Image–Point Cloud Instance Matching †

**DOI:** 10.3390/s26020718

**Published:** 2026-01-21

**Authors:** Hongxing Wang, Rui Zhu, Zelin Ye, Yaxin Li

**Affiliations:** 1 Jiangxi Provincial Key Laboratory of Precision Drive and Equipment, Jiangxi University of Water Resources and Electric Power, Nanchang 330099, China; 2 School of Mechatronics Engineering, Harbin Institute of Technology, Harbin 150001, China; 3 Fujian Key Laboratory of Intelligent Operation and Maintenance Robot Technology, Fujian (Quanzhou) Institute of Advanced Manufacturing Technology, Quanzhou 362000, China

**Keywords:** multimodal sensor fusion, instance segmentation, cross-modal perception, 3D object detection, multi-object tracking, Kalman filtering, robotic perception systems

## Abstract

Effectively fusing the rich semantic information from camera images with the high-precision geometric measurements provided by LiDAR point clouds is a key challenge in mobile robot environmental perception. To address this problem, this paper proposes a highly extensible instance-aware fusion framework designed to achieve efficient alignment and unified modeling of heterogeneous sensory data. The proposed approach adopts a modular processing pipeline. First, semantic instance masks are extracted from RGB images using an instance segmentation network, and a projection mechanism is employed to establish spatial correspondences between image pixels and LiDAR point cloud measurements. Subsequently, three-dimensional bounding boxes are reconstructed through point cloud clustering and geometric fitting, and a reprojection-based validation mechanism is introduced to ensure consistency across modalities. Building upon this representation, the system integrates a data association module with a Kalman filter-based state estimator to form a closed-loop multi-object tracking framework. Experimental results on the KITTI dataset demonstrate that the proposed system achieves strong 2D and 3D detection performance across different difficulty levels. In multi-object tracking evaluation, the method attains a MOTA score of 47.8 and an IDF1 score of 71.93, validating the stability of the association strategy and the continuity of object trajectories in complex scenes. Furthermore, real-world experiments on a mobile computing platform show an average end-to-end latency of only 173.9 ms, while ablation studies further confirm the effectiveness of individual system components. Overall, the proposed framework exhibits strong performance in terms of geometric reconstruction accuracy and tracking robustness, and its lightweight design and low latency satisfy the stringent requirements of practical robotic deployment.

## 1. Introduction

With the rapid advancement of deep learning, object detection [1], instance segmentation [2], and multimodal perception techniques [3] have achieved substantial progress in the field of robotic perception. Image-based detection methods are particularly effective in capturing rich semantic information, while LiDAR sensors provide highly accurate three-dimensional geometric structures. Effectively integrating these complementary modalities while maintaining real-time performance and system stability remains one of the core challenges in robotic perception systems [4,5].

In practical robotic deployments, perception accuracy alone is insufficient. System deployability, computational efficiency, and operational stability are equally critical, especially in dynamic multi-object environments. In such scenarios, cross-modal misalignment, frequent occlusions, and trajectory fragmentation often lead to significant performance degradation. Consequently, achieving tight semantic–geometric coupling and maintaining stable multi-object perception and tracking without resorting to overly complex network architectures constitutes an important and practically relevant system-level problem [6,7].

Motivated by these considerations, this paper proposes an instance-level image–LiDAR fusion framework for real-time robotic perception. Rather than pursuing complex feature-level fusion or end-to-end learning architectures, the proposed approach treats instance segmentation as the core carrier for cross-modal alignment. Through geometric projection, instance-level semantic information from the image domain is mapped into the LiDAR point cloud space, enabling explicit semantic–geometric coupling at the object level. Building upon this representation, lightweight point cloud clustering and classical state estimation techniques are integrated to form a complete detection and multi-object tracking pipeline. Unlike many existing projection-based fusion pipelines that rely on coarse 2D detections or heuristic proposal filtering, the proposed framework uses instance segmentation as the primary carrier for cross-modal alignment. This design enables object-level semantic consistency during projection and clustering, reducing background interference and improving association stability without introducing heavy fusion backbones. Moreover, the integration of lightweight geometric clustering with classical tracking is carefully designed to balance robustness and computational efficiency, rather than serving as a mere modular combination.

The primary contribution of this work does not lie in introducing new fundamental algorithmic components, but in systematically validating a practically deployable cross-modal perception paradigm under realistic engineering constraints.Through careful module integration and parameter design, the proposed framework achieves stable multi-object perception and tracking while maintaining computational efficiency, making it suitable for deployment on mobile robotic platforms and embedded perception systems.

## 2. Related Work

### 2.1. Image-Based Object Detection and Multi-Object Tracking

Image-based object detection and tracking have been extensively studied in the computer vision community [8]. With the rapid development of deep learning, object detection methods have evolved from traditional hand-crafted feature–based approaches to end-to-end deep neural network models [9]. Two-stage detectors, such as Faster R-CNN [10], generally achieve high detection accuracy, while single-stage detectors, including SSD [11], the YOLO family [12], and their variants, offer favorable trade-offs between accuracy and computational efficiency. Owing to their real-time capability and ease of deployment, these single-stage methods have been widely adopted in autonomous driving, intelligent surveillance, and robotic perception systems.

Instance segmentation techniques further enhance visual perception by providing pixel-level semantic information, enabling more precise characterization of object contours and local structures [13]. This capability is particularly beneficial for tasks requiring fine-grained object delineation and instance-level reasoning.

In the context of multi-object tracking (MOT), most mainstream approaches follow a tracking-by-detection paradigm, where objects are first detected independently in each frame and subsequently associated over time through data association strategies [14,15]. In recent years, transformer-based end-to-end tracking models have introduced temporal modeling mechanisms and achieved strong performance on public video benchmarks [15,16]. However, such methods typically rely on complex network architectures and large-scale training data, resulting in substantial computational overhead [17,18]. As a consequence, their direct deployment on real-time robotic platforms remains challenging.

Despite the strong semantic representation and category discrimination offered by image-based methods, the absence of explicit geometric and depth information poses inherent limitations. Challenges persist in accurate three-dimensional localization, object scale estimation, and maintaining robustness in complex dynamic environments. In particular, occlusion, illumination variation, and rapid motion can significantly degrade the stability of purely vision-based perception systems, limiting their applicability in high-reliability robotic tasks.

### 2.2. LiDAR-Based Object Detection and Tracking

LiDAR sensors provide direct access to three-dimensional spatial structure and therefore offer inherent advantages in object localization and geometric modeling. Early LiDAR-based object detection and tracking approaches primarily relied on geometric features and rule-based modeling, employing techniques such as ground segmentation, Euclidean clustering, and bounding box fitting [19,20]. These methods are characterized by clear structure and strong interpretability, and they remain practical in scenarios with limited computational resources. However, they are often sensitive to parameter tuning in complex environments and exhibit limited capability in discriminating object categories.

With the advancement of deep learning for three-dimensional point cloud processing, learning-based LiDAR perception methods have become a major research focus [21]. By leveraging voxelization, point-wise feature learning, or sparse convolution, these approaches perform high-dimensional representation learning on point cloud data and achieve substantial improvements in detection accuracy and robustness [22,23]. Correspondingly, multi-object tracking frameworks have increasingly incorporated learned feature representations to enhance data association stability [24].

Nevertheless, deep learning–based point cloud perception methods typically depend on complex network architectures and precise sensor calibration procedures, imposing stringent requirements on data quality and system synchronization. In practical engineering applications, point cloud calibration, annotation, and model training often entail considerable development effort and cost. Moreover, achieving real-time performance on embedded or resource-constrained platforms remains a significant challenge, which limits the widespread adoption of such methods in mobile robotic systems.

### 2.3. Camera–LiDAR Fusion for Object Detection and Multi-Object Tracking

To exploit the complementary strengths of visual semantic information and LiDAR-based geometric measurements, a wide range of camera–LiDAR fusion approaches have been proposed for object detection and multi-object tracking [25,26]. Depending on the fusion stage, these methods are commonly categorized into early fusion, middle fusion, and late fusion strategies. Early fusion integrates multimodal information at the feature level and can fully exploit cross-modal cues, but it typically demands precise synchronization and substantial computational resources. Late fusion adopts simpler architectures but may suffer from limited semantic consistency and spatial alignment. Middle fusion offers a compromise between performance and complexity.

In recent years, fusion methods based on bird’s-eye-view (BEV) representations [27,28] and cross-modal transformers [29] have demonstrated strong performance in autonomous driving scenarios. However, their large model size and high computational complexity make them difficult to deploy in robotic systems with strict real-time constraints. In contrast, geometry-based fusion approaches leverage the spatial calibration relationship between cameras and LiDAR sensors to establish cross-modal correspondences at the instance or object level [30,31]. Such methods feature clear structure, low implementation cost, and strong interpretability, making them attractive for practical robotic applications.

For multi-object tracking, fusion-based perception systems typically combine classical state estimation and data association techniques to maintain identity consistency and trajectory continuity. Nonetheless, in complex dynamic environments, factors such as cross-modal misalignment, occlusion, and sensor asynchrony can still undermine system stability. Therefore, achieving robust camera–LiDAR fusion for object detection and multi-object tracking, while satisfying real-time and engineering feasibility requirements, remains an important and active research problem.

## 3. Methods

**Figure 1:**
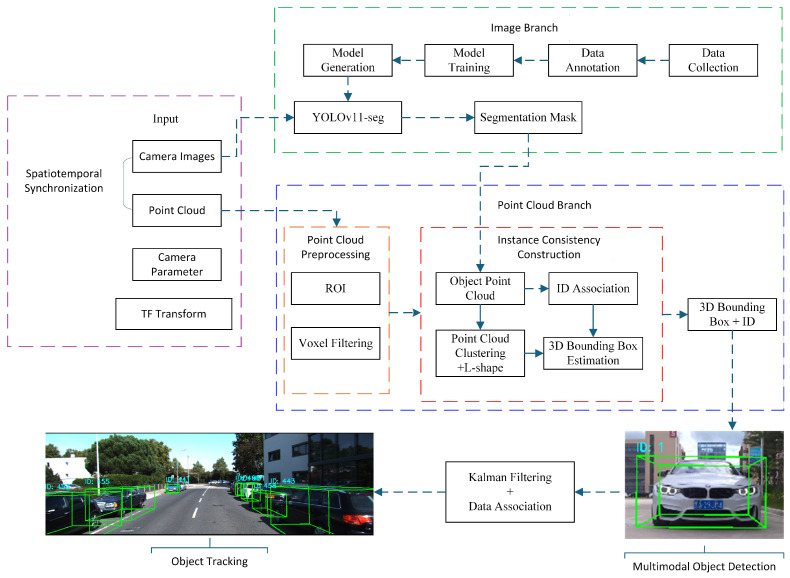
Overall workflow of the proposed instance-aware image–LiDAR fusion framework for 3D detection and multi-object tracking.

To fully exploit the complementary strengths of visual semantics and LiDAR geometry, this work proposes an instance-aware image–LiDAR fusion framework for 3D object detection and tracking. The overall pipeline consists of two coordinated branches—an image-processing stream and a point-cloud-processing stream—followed by a unified fusion and tracking module, as illustrated in Figure 1.

(1) Image branch. RGB images are processed by the YOLOv11 instance-segmentation network to produce 2D object masks and category labels, providing fine-grained semantic cues in the image domain.

(2) Point-cloud branch. Synchronized LiDAR scans undergo spatial filtering and ROI cropping. The 2D instance masks are then projected into the LiDAR space via calibrated extrinsics, enabling object-specific point-cluster extraction with reduced computation.

(3) Fusion and detection. Euclidean clustering and 3D bounding-box fitting are applied to each object-level point cluster. The reconstructed 3D boxes are back-projected onto the image plane, forming tightly coupled semantic–geometric detections.

(4) Tracking module. The fused 3D detections are fed into a Kalman-filter–based motion estimator, while a residual-weighted gating mechanism performs data association, track initialization, and life-cycle management. This enables stable multi-object tracking under occlusions and dynamic scene changes.

Through the above components, the system realizes a unified 2D–3D perception and tracking framework with strong robustness and real-time performance.

### 3.1. Image Instance Segmentation

Instance segmentation is a fundamental task in computer vision that classifies all objects within an image while delineating their contours with pixel-level precision. YOLOv11, as an advanced real-time instance segmentation framework, extends single-stage object detection by integrating segmentation directly into a unified architecture. Unlike earlier two-stage approaches, YOLOv11 employs parallel processing branches that simultaneously predict bounding boxes and generate high-quality masks associated with each detected instance. This end-to-end design enables efficient and accurate segmentation, which is essential for applications requiring fine-grained spatial localization.

In this study, the segmentation outputs of YOLOv11 are filtered according to two core criteria: (1) predictions with confidence scores below a predefined threshold are discarded to ensure reliability; and (2) the remaining results are sorted in descending order of confidence, with the number of processed instances limited by a predefined maximum to maintain high computational efficiency during multimodal fusion.

### 3.2. Image–LiDAR Fusion Detection

Given the inherent limitations of 2D imagery in perceiving object depth and scale, we propose a projection-based image–LiDAR fusion method that aligns instance-level semantic information from images with three-dimensional LiDAR geometry. The 2D masks generated by the instance segmentation network are projected into the LiDAR domain through calibrated perspective transformation, allowing pixel-wise semantic cues to be associated with corresponding 3D points. This cross-modal alignment establishes an explicit correspondence between visual semantics and spatial geometry, enabling object-level fusion and 3D reasoning.

By leveraging this alignment, the proposed method significantly enhances the robot’s holistic understanding of complex road and environmental scenes while reducing redundant point-cloud processing and improving real-time performance. The fusion output provides rich semantic–geometric representations that serve as reliable input for subsequent 3D bounding-box reconstruction and multi-object tracking.

#### 3.2.1. Data Synchronization and Coordinate Calibration

To ensure cross-sensor consistency, temporal and spatial synchronization are performed before fusion. For temporal alignment, a nearest-neighbor timestamp-matching strategy pairs each camera frame with the temporally closest LiDAR scan, ensuring that both modalities correspond to the same scene instance. For spatial alignment, extrinsic calibration is conducted to determine the rigid-body transformation between the LiDAR and camera coordinate systems, expressed as

(1) $$\left[\begin{array}{c} X_{c}\\ Y_{c}\\ Z_{c} \end{array}\right]=R_{lc}\left[\begin{array}{c} X_{l}\\ Y_{l}\\ Z_{l} \end{array}\right]+T_{lc}$$

where $R_{lc}$ and $T_{lc}$ denote the rotation matrix and translation vector, respectively, used to transform LiDAR points into the camera frame for spatial alignment.

To further reduce computational complexity, the raw point cloud is downsampled using a voxel grid filter, and only points within a defined region of interest (ROI) are retained, removing redundant data outside the effective workspace.

#### 3.2.2. 2D–3D Projection Fusion

Once temporal synchronization is completed, the aligned image–LiDAR pair is fused within a common field of view. The YOLOv11 instance-segmentation network provides pixel-level masks that serve as spatial priors on the image plane, restricting subsequent processing to semantically meaningful regions.

Given the camera intrinsic matrix *K* and the calibrated extrinsic transformation $\left(R_{lc},T_{lc}\right)$, each LiDAR point is projected onto the image plane through a standard perspective transformation:

(2) $$\left[\begin{array}{c} u\\ v\\ 1 \end{array}\right]=K\left[\begin{array}{cc} R_{lc} & T_{lc} \end{array}\right]\left[\begin{array}{c} X_{l}\\ Y_{l}\\ Z_{l}\\ 1 \end{array}\right],$$

where (u,v) denotes the pixel coordinate corresponding to the 3D LiDAR point $\left(X_{l},Y_{l},Z_{l}\right)$. Points whose projections fall within the boundaries of an instance mask are retained as belonging to that object, enabling precise extraction of object-specific 3D point clusters.

This projection-based selection process tightly couples semantic cues from the image domain with geometric structures from the LiDAR domain, yielding explicit pixel-to-point correspondences. Such alignment provides a reliable foundation for downstream 3D clustering, bounding-box reconstruction, and motion-state estimation.

#### 3.2.3. Clustering and 3D Bounding-Box Generation

After obtaining the fused point subsets corresponding to each image-domain instance, Euclidean clustering is applied to separate individual object-level point clusters according to spatial continuity. For each cluster, a 3D bounding box is estimated by fitting its geometric envelope, yielding the object’s spatial position, dimensions, and orientation. Subsequently, the reconstructed 3D bounding boxes are back-projected onto the image plane, establishing one-to-one correspondences between 2D segmented instances and their 3D geometric counterparts. This process completes the multimodal fusion detection pipeline, ensuring a tight coupling between semantic cues and spatial structure.

**Figure 2:**
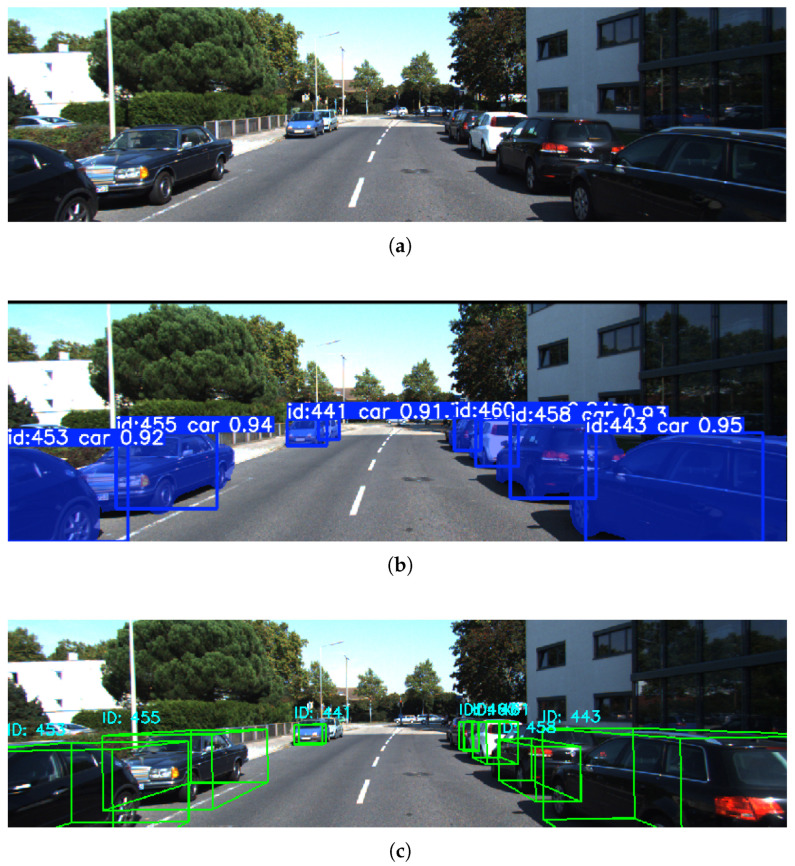
Visualization of the proposed algorithm on the KITTI dataset. (**a**) Raw input image. (**b**) Instance segmentation results. (**c**) 3D bounding-box results from LiDAR clustering.

Figure 2 provides a representative visualization of the proposed cross-modal perception framework. Figure 2a shows the raw input image; Figure 2b presents the instance segmentation results; Figure 2c illustrates the 3D bounding boxes generated from clustered LiDAR point clouds, completing the final stage of spatial localization and geometric reconstruction. Together, these results demonstrate that the proposed method achieves accurate semantic–geometric alignment across modalities, providing a reliable 3D perceptual foundation for downstream object tracking and motion-state estimation.

#### 3.2.4. Object Tracking

**Figure 3:**
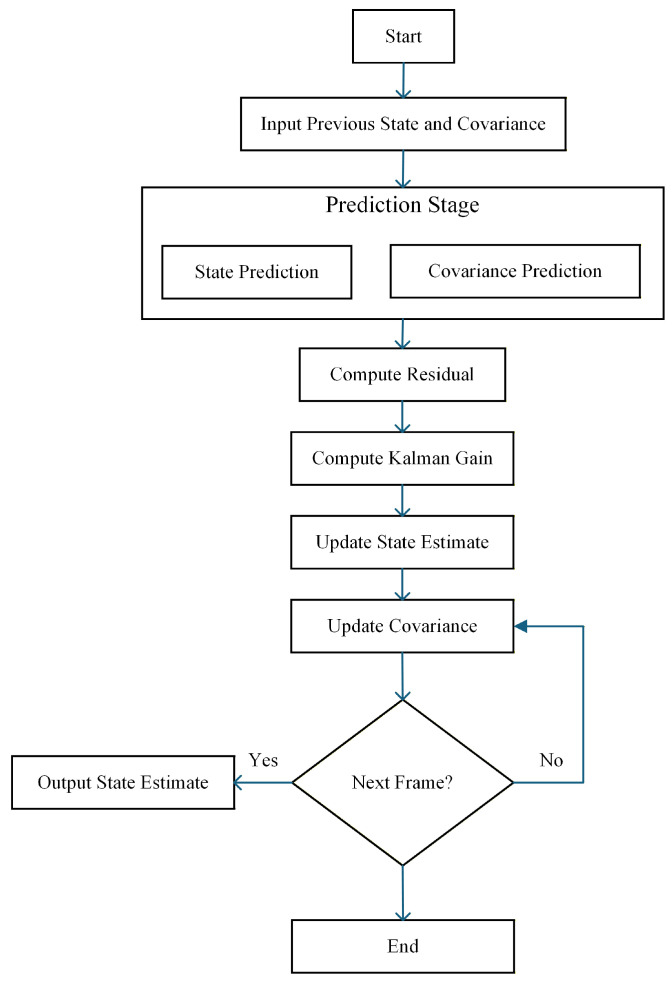
Flowchart of the Kalman filter–based state estimation process.

For robotic perception tasks, only the planar motion of ground targets is considered. The geometric center coordinates $\left(x_{0},y_{0}\right)$ of the detected objects from the fusion module are used as the measurement inputs. A constant-velocity (CV) motion model is adopted to estimate object position and velocity relative to the robot. As illustrated in Figure 3, a Kalman filter–based state estimation framework is employed.

Given a discrete time series with a sampling interval *T*, the state evolution from time *k* to k+1 is expressed as:

(3) X(k+1)=FX(k)+V(k),

where X(k) is the state vector and V(k) represents zero-mean Gaussian process noise. The state vector is defined as:

(4) $$X\left(k\right)={\left[x_{0}\left(k\right),\;{\dot{x}}_{0}\left(k\right),\;y_{0}\left(k\right),\;{\dot{y}}_{0}\left(k\right)\right]}^{T}.$$

The state transition matrix F for the constant-velocity model is given by:

(5) $$F=\left[\begin{array}{cccc} 1 & T & 0 & 0\\ 0 & 1 & 0 & 0\\ 0 & 0 & 1 & T\\ 0 & 0 & 0 & 1 \end{array}\right].$$

The measurement model is represented as:

(6) Z(k)=HX(k)+W(k),

where Z(k) denotes the measurement vector and W(k) the zero-mean Gaussian measurement noise. For planar position observations, the measurement vector and matrix are defined as:

(7) $$Z\left(k\right)={\left[x_{0}\left(k\right),\;y_{0}\left(k\right)\right]}^{T},\;H=\left[\begin{array}{cccc} 1 & 0 & 0 & 0\\ 0 & 0 & 1 & 0 \end{array}\right].$$

In the prediction stage, the prior estimates of the state mean and covariance are given by:

(8) $$\hat{X}\left(k+1|k\right)=F\;\hat{X}\left(k|k\right),$$

(9) $$P(k+1|k)=F\;P\left(k|k\right)\;F^{T}+Q,$$

where Q denotes the process noise covariance.

After receiving a new measurement, the innovation and its covariance are computed as:

(10) $$\tilde{Z}\left(k+1\right)=Z\left(k+1\right)-H\hat{X}\left(k+1|k\right),$$

(11) $$S(k+1)=HP\left(k+1|k\right)H^{T}+R,$$

where R is the measurement noise covariance. The Kalman gain and updated state estimates are obtained as:

(12) $$\hat{X}\left(k+1|k+1\right)=\hat{X}\left(k+1|k\right)+K\left(k+1\right)\tilde{Z}\left(k+1\right),$$

(13) P(k+1|k+1)=(I−K(k+1)H)P(k+1|k),

(14) $$K(k+1)=P\left(k+1|k\right)H^{T}S^{-1}\left(k+1\right),$$

where I is the identity matrix.

The process noise covariance Q and measurement noise covariance R are defined as:

(15) $$Q=\left[\begin{array}{cc} 0.5T^{2} & 0\\ T & 0\\ 0 & 0.5T^{2}\\ 0 & T \end{array}\right]\left[\begin{array}{cc} \sigma_{qx}^{2} & 0\\ 0 & \sigma_{qy}^{2} \end{array}\right]{\left[\begin{array}{cc} 0.5T^{2} & 0\\ T & 0\\ 0 & 0.5T^{2}\\ 0 & T \end{array}\right]}^{T},\;R=\left[\begin{array}{cc} \sigma_{rx}^{2} & 0\\ 0 & \sigma_{ry}^{2} \end{array}\right].$$

In this work, Q is constructed using a standard discretized white-noise acceleration model under the constant-velocity assumption, where $\left(\sigma_{qx}^{2},\sigma_{qy}^{2}\right)$ represent acceleration variances along the planar axes. Since the evaluated scenarios primarily involve ground targets with relatively smooth motion, Q is kept fixed throughout the experiments to reduce the number of free parameters and ensure stable filter behavior.

By contrast, the measurement noise covariance R directly reflects the uncertainty of LiDAR-based geometric center estimation and has a more pronounced impact on data association and tracking stability. Therefore, the sensitivity of R is explicitly evaluated through ablation experiments in Section 4.

#### 3.2.5. Multi-Object Data Association

**Figure 4:**
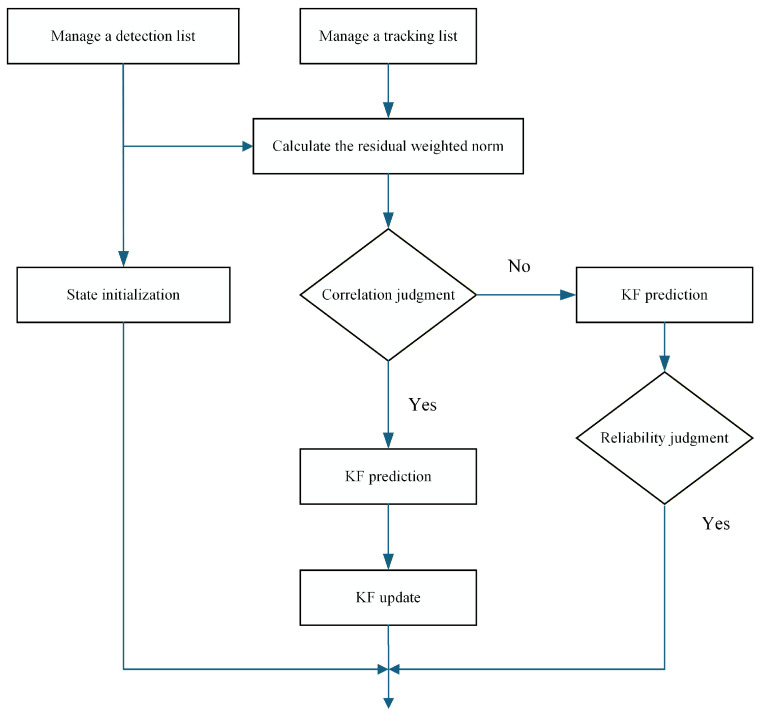
Flowchart of the multi-object data association and tracking management algorithm.

In contrast to single-target tracking, multi-object tracking requires consistent identification and maintenance of object identities across frames. At each iteration, the system maintains a set of active tracks and a set of newly observed detections. The association process integrates statistical gating, correlation evaluation, prediction, and update operations, as illustrated in Figure 4.

For each predicted track, a statistical gating operation is performed based on the Mahalanobis distance between the predicted measurement and each candidate detection. The gating criterion is defined as:

(16) $$d^{2}={\left(Z-H\hat{X}\right)}^{T}S^{-1}\left(Z-H\hat{X}\right)\leq \gamma ,$$

where $d^{2}$ denotes the residual-weighted distance, S is the innovation covariance, and γ is the gating threshold.

The threshold γ is selected according to the $\chi^{2}$ distribution with degrees of freedom equal to the measurement dimension. This statistical formulation ensures that the gating region adapts to the predicted uncertainty encoded in S and provides a principled trade-off between association strictness and robustness to measurement noise. To analyze the sensitivity of the association process to γ, different threshold values are evaluated in the ablation study presented in Section 4.

If multiple detections lie within a track’s gate, the detection yielding the smallest Mahalanobis distance is selected for association. If no detection satisfies the gating criterion, the track undergoes only the prediction step. Tracks that fail to obtain valid associations for a predefined number of consecutive frames are terminated, while unmatched detections initiate new tracks with the following initial state and covariance:

(17) $$\begin{array}{cc} \hat{X}\left(1|1\right) & =\left(z_{1}\left(1\right)\;\frac{z_{1}\left(1\right)-z_{1}\left(0\right)}{T},\;z_{2}\left(1\right)\;\frac{z_{2}\left(1\right)-z_{2}\left(0\right)}{T}\right), \end{array}$$

(18) $$\begin{array}{cc} P(1|1) & =\left[\begin{array}{cccc} \sigma_{rx}^{2} & \sigma_{rx}^{2}/T & 0 & 0\\ \sigma_{rx}^{2}/T & 2\sigma_{rx}^{2}/T^{2} & 0 & 0\\ 0 & 0 & \sigma_{ry}^{2} & \sigma_{ry}^{2}/T\\ 0 & 0 & \sigma_{ry}^{2}/T & 2\sigma_{ry}^{2}/T^{2} \end{array}\right], \end{array}$$

where the measurement vector is defined as:

(19) $$Z\left(k\right)=\left[\begin{array}{c} z_{1}\left(k\right)\\ z_{2}\left(k\right) \end{array}\right]=\left[\begin{array}{c} x_{0}\left(k\right)\\ y_{0}\left(k\right) \end{array}\right],$$

and $\left(z_{1},z_{2}\right)$ denote the observed position components along the *x*- and *y*-axes.

This association mechanism ensures robust trajectory continuity and accurate motion estimation, even under frequent occlusions and dense multi-object interactions. By combining statistically principled gating with motion-consistent updates, the framework achieves reliable identity preservation and stable performance in complex dynamic scenes.

## 4. Experiments

This section presents a comprehensive evaluation of the proposed camera–LiDAR fusion framework from both benchmark accuracy and practical deployment perspectives. The experimental design assesses not only detection and tracking performance under standardized conditions, but also runtime behavior and robustness in realistic robotic scenarios.

Experiments on the KITTI dataset are conducted to quantitatively evaluate 3D detection and multi-object tracking performance using established metrics, including 3D Average Precision (AP), CLEAR MOT, and HOTA.In addition, real-world experiments are performed on a mobile computing platform operating under battery-powered constraints to validate online performance, system stability, and deployability. It is emphasized that the objective of this evaluation is not to compete with fully supervised LiDAR-based 3D detection networks, but rather to demonstrate that an instance-aware geometric fusion pipeline requiring no point cloud annotation or training can achieve a practical balance between accuracy, robustness, and real-time performance on resource-constrained robotic platforms.

### 4.1. Experiments on the KITTI Dataset

#### 4.1.1. Experimental Setup and Training Protocol

All benchmark experiments are conducted on the KITTI dataset. Offline evaluations are performed on a workstation running Ubuntu 20.04 with ROS Noetic, with the hardware configuration summarized in Table 1.

For the image-domain instance segmentation module, training is performed on the KITTI MOTS dataset, which consists of 21 annotated sequences. Sequences 0000–0015 are used for training, while sequences 0016–0020 are reserved for validation. All experimental results reported in this section are obtained exclusively on the validation sequences, ensuring that no evaluation frames are included in the training process.

The YOLOv11n-seg model is adopted as the base instance segmentation network and trained using the parameters listed in Table 2. The resulting model achieves the validation performance shown in Table 3, providing reliable 2D detection and instance-level segmentation priors for subsequent image–LiDAR fusion.

#### 4.1.2. 3D Detection Performance

We chose to test the 3D Average Precision (AP) of the proposed algorithm on the validation set. Unlike fully supervised LiDAR-based 3D detectors that rely on dense point cloud annotations and deep 3D networks, the proposed method reconstructs 3D bounding boxes through geometric fitting guided by 2D instance masks. Consequently, its performance characteristics differ fundamentally from those of learning-based approaches, particularly under strict IoU thresholds.

The detection, clustering, and tracking parameters used in this experiment are summarized in Table 4, and the quantitative detection results are reported in Table 5.

The proposed method achieves an AP_3*D*_ of 40.06% on the Easy subset and 22.83% on the Moderate subset for the Car category on the validation set. Although these results remain below those reported by fully supervised 3D detection networks, they demonstrate clear practical value for a perception pipeline that requires neither 3D point cloud annotations nor dedicated 3D detection network training.

The performance degradation observed from Easy to Hard cases highlights the inherent dependence of geometric fitting accuracy on LiDAR point density. At longer sensing ranges, sparse point returns limit the precision with which object boundaries and spatial extents can be estimated. Moreover, the KITTI evaluation protocol imposes a strict IoU threshold of 0.7 for the car category, such that even minor geometric misalignments—for example, IoU values in the range of 0.5–0.6 are heavily penalized. This characteristic explains the performance gap between geometry-based methods and fully learning-based approaches under the KITTI evaluation metric.

To validate the effectiveness of using instance segmentation masks in this paper as compared to solely relying on 2D detection boxes, we conducted a 3D Average Precision (AP) experiment without masks on the validation set using the same parameters. The experimental results are shown in Table 6.

The results shown in Table 6 indicate significant differences in both BEV Detection (AP_*BEV*_) and 3D Detection (AP_3*D*_) between the cases with and without masks for both categories. In the Car category, the 3D AP decreased from 40.06% to 32.35% under Easy difficulty and from 15.85% to 13.39% under Hard difficulty when no masks were used. In the Pedestrian category, the 3D AP decreased from 27.27% to 21.63% under Easy difficulty and from 7.35% to 3.54% under Hard difficulty when no masks were used.

The reasons for these results can be summarized as follows:

1. **3D Detection for the Car Category**: At medium-to-close range with dense occlusions, the 3D localization of vehicles becomes more challenging. When no masks are used, the YOLO 2D detection boxes are more likely to mix irrelevant point clouds with the target, leading to a significant drop in 3D AP.
2. **Challenges in the Pedestrian Category**: Due to the small size of pedestrian targets and their susceptibility to occlusion, even though the accuracy of 2D detection boxes is relatively high, the occlusions and small target size make 3D localization significantly harder, resulting in very low 3D AP under Hard difficulty.
3. **Importance of Masks**: The masks provide more accurate 2D bounding boxes, which aid in improving the accuracy of 3D localization. Especially in cases with small targets and severe occlusion, the masks effectively enhance the accuracy of 3D detection.

#### 4.1.3. Multi-Object Tracking Performance

To validate the multi-object tracking (MOT) performance of the proposed algorithm, we conducted experimental evaluations on the validation set, with experimental parameters set consistent with those in Table 4. To thoroughly verify the algorithm’s effectiveness, we designed three comparative schemes:

1. Vision-only scheme: This baseline combines the YOLO-seg model used in this paper with the ByteTrack algorithm, relying solely on image data;
2. LiDAR-only scheme: This variant relies exclusively on point cloud data. It applies category-specific dimensional constraints and performs clustering, geometric fitting, and tracking within the proposed framework;
3. Mask-free fusion scheme: This variant utilizes the proposed fusion framework but relies solely on 2D bounding boxes instead of instance segmentation masks for data association.

The experimental results are shown in Table 7.

As shown in Table 7, the experimental results demonstrate that the proposed fusion algorithm exhibits significant advantages in tracking stability in complex scenarios.

First, in terms of key metrics that reflect tracking continuity and identity consistency, the proposed algorithm performs the best. Compared to the pure visual (Only Camera) scheme, the proposed method improves IDF1 by 4.54% (from 68.25% to 72.79%), while the number of identity switches (IDSW) significantly decreases by 22.8% (from 35 to 27). This result clearly proves that the introduction of 3D point cloud geometry and depth information effectively compensates for the limitations of 2D vision in handling target occlusion and interaction, thereby maintaining more robust target trajectories. In contrast, the pure LiDAR (Only Lidar) scheme, due to the sparse point clouds and lack of texture semantics, leads to a large number of false detections (with IDSW as high as 42) and missed detections, with all metrics performing poorly and struggling to handle complex environments independently.

Second, the critical role of instance segmentation masks in fine-grained fusion is further confirmed. Compared to the scheme that uses only detection boxes for point cloud cropping (Ours no mask), the introduction of masks (Ours) leads to an improvement in precision from 82.46% to 86.39%, with IDF1 also increasing by 2.76%. This indicates that in crowded scenarios such as intersections, simple rectangular bounding box fusion tends to introduce background noise or point cloud interference from adjacent vehicles, while the mask effectively removes these interferences, ensuring the purity of 3D state estimation.

Finally, regarding the trade-off in detection quality, the proposed algorithm adopts a strategy that prioritizes precision over recall. Although the strict image-mask-point cloud matching mechanism results in a slight decrease in Recall (from 63.54% to 60.25%) compared to the vision-only scheme, leading to a minor drop in MOTA (43.48%), it effectively filters out background false positives inherent in visual detection. This strategy, sacrificing partial recall for higher precision (86.39%) and stability (IDF1 72.79%), ensures that every detected object possesses high confidence and accurate 3D spatial positioning. This is of greater practical significance for the safe navigation and obstacle avoidance of mobile robots than merely pursuing the quantity of detections.

#### 4.1.4. Ablation Studies

To analyze the contribution of individual components and parameter choices, extensive ablation studies are conducted on the validation set, with the results summarized in Table 8. The ablation experiments evaluate the clustering distance thresholds, minimum cluster size, Mahalanobis gating threshold γ, and measurement noise covariance R.

Table 8 summarizes the ablation results for the Car category. Overall, the proposed method achieves the best balance across MOTA, IDF1, and HOTA, whereas other variants maximize individual metrics at the expense of global performance.

The clustering distance threshold presents a trade-off between recall and stability. A strict threshold (0.1) improves Precision but causes over-segmentation, while enlarging it to 0.7 leads to object adhesion, significantly increasing identity switches. The selected 0.5 yields the most balanced performance.

The minimum cluster size governs noise suppression versus completeness. A small threshold (10 points) introduces noisy detections, degrading Precision and increasing IDSW despite high Recall. Conversely, an excessive threshold (2000 points) filters out distant targets, notably reducing Recall.

The gating threshold γ controls association strictness. Lower values reduce identity switches via conservative matching but may reject valid associations, while higher values improve recall at the cost of false matches. The proposed configuration compromises between these competing effects.

Finally, measurement noise *R* determines filter sensitivity. A small *R* (0.03) amplifies geometric jitter, increasing identity switches (30). While increasing *R* to 0.1 minimizes IDSW (25) and smooths trajectories, it induces state lag that reduces Recall and IDF1 compared to the optimal setting (R=0.05), which effectively balances stability with dynamic responsiveness.

### 4.2. Real-World Experiments

#### 4.2.1. Experimental Setup

To evaluate the runtime performance of the proposed framework in practical engineering scenarios, the model described in Table 3 and the parameter settings listed in Table 4 are adopted. Experiments are conducted to assess single-object detection accuracy, the runtime latency of individual modules, and overall system stability. The hardware and software configuration of the workstation, as well as the sensor specifications used during the experiments, are summarized in Table 9.

#### 4.2.2. Single-Target Detection and Tracking Experiment

The single-target experiment took place in a 30m×30m open test field, where the robot detected and tracked a single vehicle. The vehicle measured 4803 mm in length, 1887 mm in width, and 1404 mm in height. It moved through the field in both longitudinal and lateral directions at speeds of 5 km/h and 15 km/h, allowing evaluation under different motion patterns.

**Figure 5:**
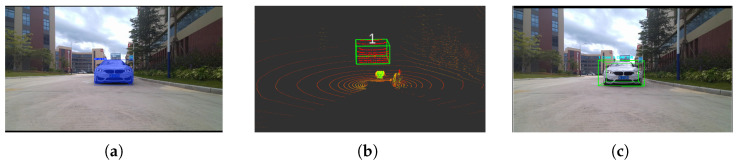
Cross-modal perception results under the longitudinal scenario. (**a**) Instance segmentation results. (**b**) LiDAR-domain point clusters. (**c**) 3D bounding-box generation.

**Figure 6:**
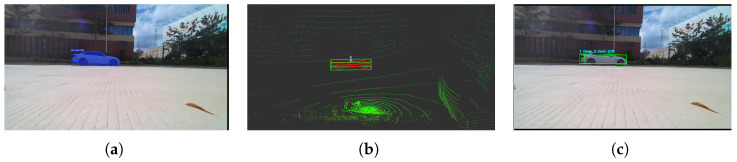
Cross-modal perception results under the horizontal scenario. (**a**) Instance segmentation results. (**b**) LiDAR-domain point clusters. (**c**) 3D bounding-box generation.

Figure 5 and Figure 6 illustrate representative detection and tracking results for two motion conditions. Scenario 1 presents the case where the vehicle moves longitudinally across the field, while Scenario 2 demonstrates lateral motion. In both cases, the proposed method successfully fuses the semantic cues from image-domain instance masks with geometric constraints from LiDAR point clouds, achieving stable and continuous tracking throughout the trajectory. These results verify the effectiveness of the framework in single-target scenarios and establish a baseline for subsequent multi-target evaluations.

As shown in the figures, the proposed fusion-based detection method is capable of extracting the 3D geometric attributes of the target from LiDAR point clouds. The statistical results are summarized in Table 10, where each value represents the average measurement obtained under different test conditions.

As shown in Table 10, the proposed fusion-based method demonstrates stable detection performance across all test scenarios. For the 5 km/h cases, the estimated dimensions remain close to the ground-truth values, with centimeter-level deviations in length, width, and height. When the speed increases to 15 km/h, the deviations become slightly larger, which aligns with the increased motion speed, resulting in reduced point density and mild fluctuations in the reconstructed dimensions. Across all scenarios, velocity estimation in the *x*- and *y*-directions closely matches the commanded motion, indicating that the proposed system maintains high reliability in both geometric reconstruction and motion-state estimation.

#### 4.2.3. Performance on a Mobile Computing Platform

To evaluate the deployability of the proposed perception system on platforms with limited computational resources, real-world experiments were conducted using a laptop as the onboard processing unit. The evaluation sequence lasted 248 s and covered a range of representative operating conditions, including stationary vehicles, low-speed cruising, and interactions with dynamic pedestrians.

**Figure 7:**
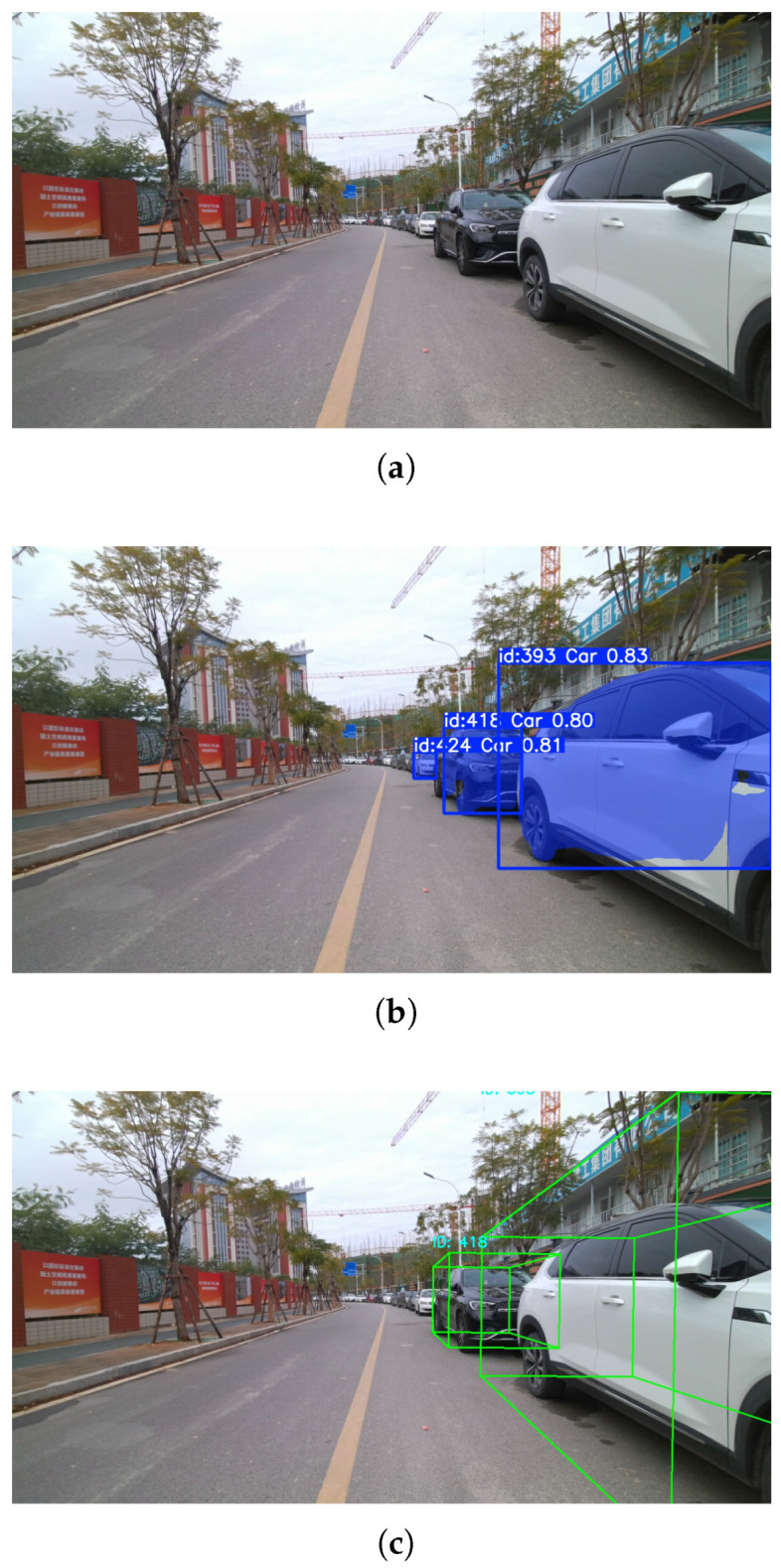
Visualization of the proposed algorithm verified in real-world scenarios. (**a**) Raw input image. (**b**) Instance segmentation results. (**c**) 3D bounding-box results from LiDAR clustering.

As illustrated in Figure 7, the test environment features dense traffic and frequent occlusions, posing practical challenges for online perception and tracking. Detailed performance statistics for each processing stage are reported in Table 11. Since the experiments were performed in a fully online setting, a latest-frame-first strategy was applied to the YOLO inference node by setting the message queue size to 1. This configuration prevents image buffer accumulation and avoids latency buildup caused by delayed frame processing, thereby ensuring stable real-time operation during prolonged execution.

Table 11 presents the runtime performance statistics of the multi-sensor perception system proposed in this paper under online load conditions. The statistics cover the average latency, worst-case latency, frame drop rate, and deadline miss rate of each key module under a given input frequency. Experimental results indicate that each functional module of the system achieves high data throughput stability while ensuring computational efficiency. Specifically, the target detection module, as the main computational bottleneck, achieves an average processing delay of 69.7 ms (standard deviation of 8.6 ms), with the maximum latency controlled within 154.2 ms. The sensor fusion and target tracking modules achieve average latencies of 50.3 ms and 1.0 ms, respectively. Notably, the small frame drop rates observed within each module (0.9% for the detection module and 0.5% for the fusion and tracking module) are not caused by computational bottlenecks, but rather due to the soft time synchronization mechanism employed by the system. To ensure strict temporal alignment between LiDAR point clouds and camera images, the system actively filters out frames with timestamp deviations exceeding the threshold, thereby ensuring the accuracy of multi-modal data fusion.

Regarding the system’s end-to-end performance, the overall average latency is 120.8 ms, with the worst-case latency reaching 181.9 ms. The 33.3% system-level frame drop rate shown in the data is an inherent result of multi-sensor frequency alignment. Specifically, the system down-samples the 15 Hz visual input stream to match the 10 Hz LiDAR input stream, indicating that the system effectively processes all available synchronized frames without blocking due to insufficient computing power. More critically, the reliability assessment of the real-time system shows that under a 150 ms soft deadline, only 5.4% of the frames miss their deadlines, while the violation rate drops further to 0.1% under a 180 ms hard deadline. This demonstrates that in 99.9% of the test conditions, the system consistently completes the full computation—from sensor input to trajectory output—within 180 ms. These results fully validate the robustness of the system when handling online loads and complex scenarios, meeting the stringent real-time requirements of mobile robots’ hard real-time perception systems.

## 5. Discussion

The experimental results presented in Section 4 demonstrate that the proposed fusion-based detection and multi-object tracking framework achieves robust performance in both controlled robotic experiments and complex dynamic scenarios from the KITTI dataset. By effectively combining the semantic richness of image data with the geometric precision of LiDAR point clouds, the system significantly reduces false positives compared to single-modality baselines. Furthermore, the residual-weighted gating mechanism ensures stable trajectory management, maintaining consistent IDs even in cluttered environments.

### 5.1. Limitations

Despite the promising results, we acknowledge several limitations in the current framework, which primarily stem from the engineering trade-offs made to ensure real-time performance on embedded platforms:

1. Motion Model Simplification: As noted in the methodology, our tracking module relies on a linear Constant Velocity (CV) model. While this choice minimizes computational overhead and is effective for smooth motions at high frame rates, it theoretically limits the system’s ability to track targets undergoing rapid acceleration or sudden directional changes. In extreme maneuvering scenarios, this may result in temporary tracking lag.
2. Assumption of Planar Motion: The system is designed specifically for ground mobile robots (UGVs) and assumes objects move on a 2D plane. This simplification allows for efficient state estimation but restricts the framework’s applicability in environments with significant vertical variations (e.g., uneven terrain) or for platforms requiring 6-DoF tracking (e.g., drones).
3. Dependence on 2D Detection: Since the fusion pipeline is proposal-based, the final performance is inherently bounded by the 2D detector (YOLOv11). In scenarios with severe lighting degradation (e.g., total darkness) or where objects are heavily occluded in the image domain, missed detections can propagate to the fusion stage.

### 5.2. Future Work

To address these challenges, future work will focus on three key directions:

- Adaptive State Estimation: We plan to investigate lightweight Adaptive Kalman Filters (AKF) or Interacting Multiple Model (IMM) algorithms. These methods can dynamically adjust noise covariance or switch motion models to better handle maneuvering targets without incurring excessive computational costs.
- Temporal and Appearance Association: To mitigate the effects of occlusion and sparse data, we aim to incorporate appearance-based Re-Identification (Re-ID) features into the data association step, allowing the system to recover tracks after long-term occlusion.

## 6. Conclusions

This paper proposes a detection method that integrates image-based instance segmentation with LiDAR point cloud clustering and geometric fitting. The overall framework is concise and easy to implement and extend from an engineering perspective, making it well suited for mobile robotic applications. By effectively combining the rich semantic information provided by camera images with the precise spatial geometry captured by LiDAR sensors, the proposed method enables three-dimensional detection of objects of interest, including the estimation of their positions and physical dimensions.

Building upon this fusion framework, a complete multi-object tracking system is further developed. A tracking gate mechanism is designed to support multi-target data association, new object state initialization, and track lifecycle management, enabling reliable estimation of object velocities and stable tracking of multiple dynamic targets over time.

To comprehensively evaluate the proposed approach, experiments were first conducted on the KITTI dataset, where both three-dimensional average precision (3D AP) and multi-object tracking accuracy (MOTA) were assessed, demonstrating favorable detection and tracking accuracy. Subsequently, real-world experiments were performed on a mobile computing platform to examine practical deployment performance. The results indicate that the proposed method maintains competitive 3D detection accuracy while exhibiting low computational overhead and high information utilization efficiency, thereby satisfying real-time operational requirements.

Overall, the proposed approach demonstrates strong practical value for robotic perception tasks. Owing to its lightweight design and robust real-time performance, the framework is particularly suitable for deployment on mobile and embedded robotic platforms with limited computational resources.

## Figures and Tables

**Table 1:** Software and hardware configuration of the workstation.

Component	Specification
CPU	Intel Core i5-13400F (Intel Corp., Santa Clara, CA, USA)
GPU	NVIDIA GeForce RTX 3070 8GB (NVIDIA Corp., Santa Clara, CA, USA)
RAM	32 GB
Operating system version	Ubuntu 20.04
ROS Version	Noetic

**Table 2:** Training parameters of YOLOv11n-seg.pt.

Parameter	Value
Learning rate	0.01
Optimizer	SGD
IoU threshold	0.7
Mosaic	1.0
Warmup epochs	3.0
Batch size	32
Epochs	200
Input size	640

**Table 3:** Model performance obtained from training on KITTI MOTS.

Class	Box (P)	Box (R)	Box mAP50	Box mAP50–95	Mask (P)	Mask (R)	Mask mAP50	Mask mAP50–95
ALL	0.707	0.495	0.564	0.365	0.693	0.464	0.521	0.283
Car	0.728	0.712	0.757	0.570	0.728	0.683	0.729	0.451
Pedestrian	0.685	0.278	0.371	0.161	0.658	0.245	0.313	0.114

**Table 4:** YOLO parameter settings, point cloud clustering parameters, and tracking parameter configurations.

Parameter	Value
YOLO Instance Segmentation Parameters	
YOLO model	YOLOv11n-seg.pt
Confidence threshold	0.5
IoU threshold	0.45
Image size	1280
LiDAR Preprocessing and Clustering Parameters	
Voxel downsampling leaf size	0.1 m
Cluster tolerance (distance threshold)	0.3 m
Minimum cluster size	500
Maximum cluster size	10,000
Target tracking–related settings	
Gating threshold γ	6
Measurement noise *R*	0.05

**Table 5:** 3D AP detection results of the proposed method on the validation set.

Metric	Car (Easy)	Car (Mod)	Car (Hard)	Pedestrian (Easy)	Pedestrian (Mod)	Pedestrian (Hard)
2D Detection (AP 2D)	86.13%	74.10%	65.27%	81.25%	62.45%	51.03%
BEV Detection (AP BEV)	52.34%	37.28%	24.67%	44.57%	26.35%	18.34%
3D Detection (AP 3D)	40.06%	22.83%	15.85%	27.27%	12.47%	7.35%

**Table 6:** 3D AP detection results of the proposed method without using masks on the validation set.

Metric	Car (Easy)	Car (Mod)	Car (Hard)	Pedestrian (Easy)	Pedestrian (Mod)	Pedestrian (Hard)
2D Detection (AP 2D)	88.43%	77.83%	64.95%	70.25%	53.28%	48.74%
BEV Detection (AP BEV)	45.84%	36.75%	23.52%	41.36%	22.86%	15.41%
3D Detection (AP 3D)	32.35%	18.49%	13.39%	21.63%	7.46%	3.54%

**Table 7:** Comparison of Experimental Results for Multi-Object Tracking (MOT) Performance under Different Schemes.

Metric	MOTA	IDF1	HOTA	Recall (%)	Precision (%)	IDSW
Only Camera	51.74	68.25	55.58	63.54	84.84	35
Only LiDAR	32.24	53.46	29.56	39.78	62.53	42
Ours (no mask)	40.59	70.03	38.95	57.22	82.46	30
Ours	43.48	72.79	41.37	60.25	86.39	27

**Table 8:** Ablation study results of tracking parameters.

Parameter	Value	MOTA	IDF1	HOTA	Recall (%)	Precision (%)	IDSW
Cluster Tolerance	0.1	36.85	70.92	35.53	51.43	90.14	28
	0.5	42.18	71.08	40.85	61.12	83.53	34
	0.7	38.62	68.81	36.90	61.85	80.26	40
Minimum cluster size	10	33.52	66.84	32.56	61.57	78.61	45
	2000	37.92	69.86	36.22	48.27	91.35	19
Gating threshold γ	3.0	41.23	71.56	39.87	56.81	90.54	21
	9.0	40.53	69.90	38.54	61.58	83.26	36
Measurement noise *R*	0.03	41.48	70.86	39.83	59.24	85.26	30
	0.1	42.75	72.10	40.94	59.93	85.59	25
Ours (Baseline)		43.48	72.79	41.37	60.25	86.39	27

**Table 9:** Host computer software and hardware configuration and sensor parameters.

Computer Model	DELL G3 3590 (2019)
CPU	Intel Core i5-9300H (Intel Corp., Santa Clara, CA, USA)
GPU	NVIDIA GeForce GTX 1650 4GB (NVIDIA Corp., Santa Clara, CA, USA)
RAM	12 GB
Operating system version	Ubuntu 20.04
ROS Version	Noetic
Camera	Azure Kinect DK (Microsoft Corp., Redmond, WA, USA) (720P, 15 Hz)
LiDAR	RoboSense Helios-32 (RoboSense, Shenzhen, China) (10 Hz)

**Table 10:** Statistical Results of the Single-Target Experiment.

Test Scenario	Length (m)	Width (m)	Height (m)	Velocity *x* (m/s)	Velocity *y* (m/s)
Scenario 1—5 km/h	—	1.89	1.43	1.37	0.02
Scenario 1—15 km/h	—	1.91	1.45	4.10	0.05
Scenario 2—5 km/h	4.81	—	1.44	0.03	1.38
Scenario 2—15 km/h	4.85	—	1.47	0.06	4.15

**Table 11:** Performance metrics of different processing modules and system end-to-end latency.

Processing Module	Avg Latency ± Std (ms)	Max Latency (ms)	Input Freq (Hz)	Output Freq (Hz)	Frame Drop Rate (%)
Object Detection	69.7±8.6	154.2	15	15	0.9
Sensor Fusion	50.3±19.0	110.4	10	10	0.5
Object Tracking	1.0±0.4	3.7	10	10	0.5
System End-to-End	120.8±19.2	181.9	15	10	33.3
Deadline Miss Rate					
>150ms	5.4%				
>180ms	0.1%				

## Data Availability

The data presented in this study are available on request from the corresponding author.
